# Genotoxicity of multi-walled carbon nanotubes at occupationally relevant doses

**DOI:** 10.1186/1743-8977-11-6

**Published:** 2014-01-30

**Authors:** Katelyn J Siegrist, Steven H Reynolds, Michael L Kashon, David T Lowry, Chenbo Dong, Ann F Hubbs, Shih-Houng Young, Jeffrey L Salisbury, Dale W Porter, Stanley A Benkovic, Michael McCawley, Michael J Keane, John T Mastovich, Kristin L Bunker, Lorenzo G Cena, Mark C Sparrow, Jacqueline L Sturgeon, Cerasela Zoica Dinu, Linda M Sargent

**Affiliations:** 1 National Institute for Occupational Safety and Health, Morgantown, WV 26505, USA; 2 Department of Chemical Engineering, Benjamin M. Statler College of Engineering and Mineral Resources, West Virginia University, Morgantown, WV 26505, USA; 3 Department of Biochemistry, Mayo Clinic, 2001st Street NW, Rochester, MN 55905, USA; 4 Department of Occupational & Environmental Health Sciences, West Virginia University, Morgantown, WV 26506, USA; 5 RJ Lee Group Inc, 300 Hockenberg Drive, Monroeville, PA 15146, USA

## Abstract

Carbon nanotubes are commercially-important products of nanotechnology; however, their low density and small size makes carbon nanotube respiratory exposures likely during their production or processing. We have previously shown mitotic spindle aberrations in cultured primary and immortalized human airway epithelial cells exposed to single-walled carbon nanotubes (SWCNT). In this study, we examined whether multi-walled carbon nanotubes (MWCNT) cause mitotic spindle damage in cultured cells at doses equivalent to 34 years of exposure at the NIOSH Recommended Exposure Limit (REL). MWCNT induced a dose responsive increase in disrupted centrosomes, abnormal mitotic spindles and aneuploid chromosome number 24 hours after exposure to 0.024, 0.24, 2.4 and 24 μg/cm^2^ MWCNT. Monopolar mitotic spindles comprised 95% of disrupted mitoses. Three-dimensional reconstructions of 0.1 μm optical sections showed carbon nanotubes integrated with microtubules, DNA and within the centrosome structure. Cell cycle analysis demonstrated a greater number of cells in S-phase and fewer cells in the G2 phase in MWCNT-treated compared to diluent control, indicating a G1/S block in the cell cycle. The monopolar phenotype of the disrupted mitotic spindles and the G1/S block in the cell cycle is in sharp contrast to the multi-polar spindle and G2 block in the cell cycle previously observed following exposure to SWCNT. One month following exposure to MWCNT there was a dramatic increase in both size and number of colonies compared to diluent control cultures, indicating a potential to pass the genetic damage to daughter cells. Our results demonstrate significant disruption of the mitotic spindle by MWCNT at occupationally relevant exposure levels.

## Introduction

Carbon nanotubes (CNT) are used in many consumer and industrial products including electronic devices, protective clothing, sports equipment and medical devices as well as vehicles for drug delivery [1-3]. Due to the wide variety of applications, the nanotechnology industry is predicted to grow to one trillion dollars by 2015 [4]. The low density and small size of carbon nanotubes make respiratory exposure likely during production and processing. Indeed, recent investigations have shown that carbon nanotubes can be aerosolized under workplace conditions [5-8]. Although carbon nanotubes have a large variety of applications, their potential health effects have not been fully investigated.

The low density, fiber-like geometry and durability of carbon nanotubes are characteristics shared with asbestos [9,10]. Single-walled and multi-walled carbon nanotubes have been shown to enter cells and induce DNA damage, sister chromatid exchange, chromosome damage and micronuclei *in vitro* in human keratinocytes, human breast cancer cell lines, human lung cancer epithelial cells and immortalized mouse fibroblasts (Balb/3 T3 cells) [11-15]. Micronuclear formation can result from either a high level of chromosome damage or mitotic spindle disruption. Research by Di Giorgio et al., 2011 demonstrated significant chromosome breakage by analysis of chromosome spreads as well as DNA damage by the comet assay in a mouse macrophage cell line 24–48 hours after exposure to MWCNT (10–25 nm) and SWCNT (0.7-1.2 nm) material [16]. The carbon nanotube-exposed cells also had high levels of intracellular reactive oxygen species suggesting that carbon nanotubes can cause chromosome damage through reactive oxygen species [16]. Increased DNA damage due to oxygen radicals was also observed in imprinting control region mice (ICR) mice *in vivo* following intratracheal installation of 0.05 or 0.2 mg MWCNT/mouse [11]. Carbon nanotubes bind to DNA at G-C rich regions in the chromosomes including telomeric DNA [17,18]. The interaction with the DNA results in a conformational change. DNA intercalation and telomeric binding can induce chromosome breakage suggesting that interaction of the nanotubes with the DNA may also be a source of chromosome damage. Recent investigations have shown that acid-washed single-walled carbon nanotubes of 1–4 nm in diameter and one micron in length induce centrosome fragmentation, multipolar mitotic spindles and errors in chromosome number in cultured immortalized and primary lung epithelial cells [19]. Furthermore, exposure of cancer cell lines to MWCNT of 5–10 nm diameter and one micron in length also results in multipolar mitotic spindles [20].

Mitotic spindle disruption and aneuploidy are a concern because these effects have been observed with the carcinogenic fiber, asbestos. *In vitro* investigations have demonstrated that chrysotile asbestos exposure causes multipolar mitotic spindles and a G2/M block similar to SWCNT and vanadium pentoxide exposure [19,21-24]. Asbestos exposure disrupts the mitotic spindle and causes aneuploidy through amplification of the centrosome [21,22]. By contrast, the mitotic disruption and aneuploidy resulting from vanadium pentoxide and SWCNT is associated with fragmented centrosomes [19,23]. Furthermore, *in vitro* examinations of asbestos and vanadium pentoxide potency have demonstrated that the disruption of the mitotic spindle and aneuploidy in cultured cells is strongly correlated with *in vivo* carcinogenesis [25-28]. Together these investigations indicate the importance of genotoxicity in carcinogenesis as well as validating the significance of culture models to predict carcinogenesis.

To simulate aerosol exposures in the workplace, rodents have been exposed to high aspect ratio particles by inhalation, pharyngeal aspiration or intratracheal installation. In a manner similar to asbestos, rodent pulmonary exposure to biopersistant carbon nanotubes has been shown to result in lung inflammation, epithelial cell proliferation, cellular atypia and mutations in the K-ras gene [29-32]. The lung is the principal site of carbon nanotube deposition and toxicity following aspiration or inhalation [31,33]. *In vivo* investigations have demonstrated that carbon nanotube exposure can cause macrophages without nuclei as well as dividing macrophages connected by nanotubes [30,31]. Exposure of rats to the MWCNT by pharyngeal aspiration has been shown to result in micronuclei formation in Type II epithelial cells further indicating the potential for genetic damage [13]. Inflammation, cellular proliferation, cellular atypia, mitotic spindle disruption, centrosome fragmentation and errors in chromosome number are linked with the development of cancer [34-40]. Chronic exposures to asbestos particles which induce strong inflammatory, proliferative and genotoxic responses in the lung are associated with an increased incidence of lung cancer in rodents [41,42]. Although the lung is the key target organ for particle toxicity, high aspect ratio carbon nanotubes have been shown to translocate to the subpleural space indicating that the mesothelial cells are also a potential target [43,44].

The overall objective of our study was to examine the role of CNT diameter in the nanotube-induced genetic damage using carbon nanotubes prepared with the same acid washing procedure and one micron length used in our previous studies to evaluate the potential genotoxicity of the narrower SWCNT [24,45]. Because vanadium pentoxide has been demonstrated to induce aneuploidy and mitotic spindle disruption through fragmentation of the centrosome, we selected vanadium as the positive control for genotoxicity. Immortalized and primary lung epithelial cells were examined for the potential of MWCNTs to cause aneuploidy, mitotic spindle disruption, centrosome fragmentation, and cell cycle distribution following exposure of primary and immortalized human epithelial cells to occupationally relevant doses of 10–20 nm diameter MWCNT. Primary cells were used in the assays since the normal karyotype made it possible to determine changes in chromosome number after exposure. The concentrations chosen for the current investigation were selected to be relevant to previous *in vivo* exposure doses of MWCNT of 10–40 μg/mouse (0.5 μg, 1 μg, and 2 μg/kg respectively) reported by Porter et al. [30]. In brief, the mouse lung burdens per alveolar epithelial surface area of 500 cm^2^/mouse lung [46] correspond to *in vitro* concentrations of 0.02–0.08 μg/cm^2^. The minimal *in vitro* dose of 0.02 μg/cm^2^ MWCNT would require 4 weeks of exposure at the Occupational Safety and Health Administration (OSHA) permissible exposure limit for particles with an aerodynamic diameter of 5 microns or less of 5 mg/m^3^[47,48]. NIOSH has recently reduced the REL from 7 μg/m^3^ to 1 μg/m^3^[49] . Although exposure to concentrations of carbon nanotubes equivalent to the current NIOSH REL of 1 μg/m^3^ would require 34 years to yield a equivalent exposure of the 0.024 μg/cm^2^, levels of MWCNT between 0.7 and 331 μg/m^3^ have been measured in workplace air [6,7,50-52].

## Results

### Characterization of carbon nanotubes

**Figure 1:**
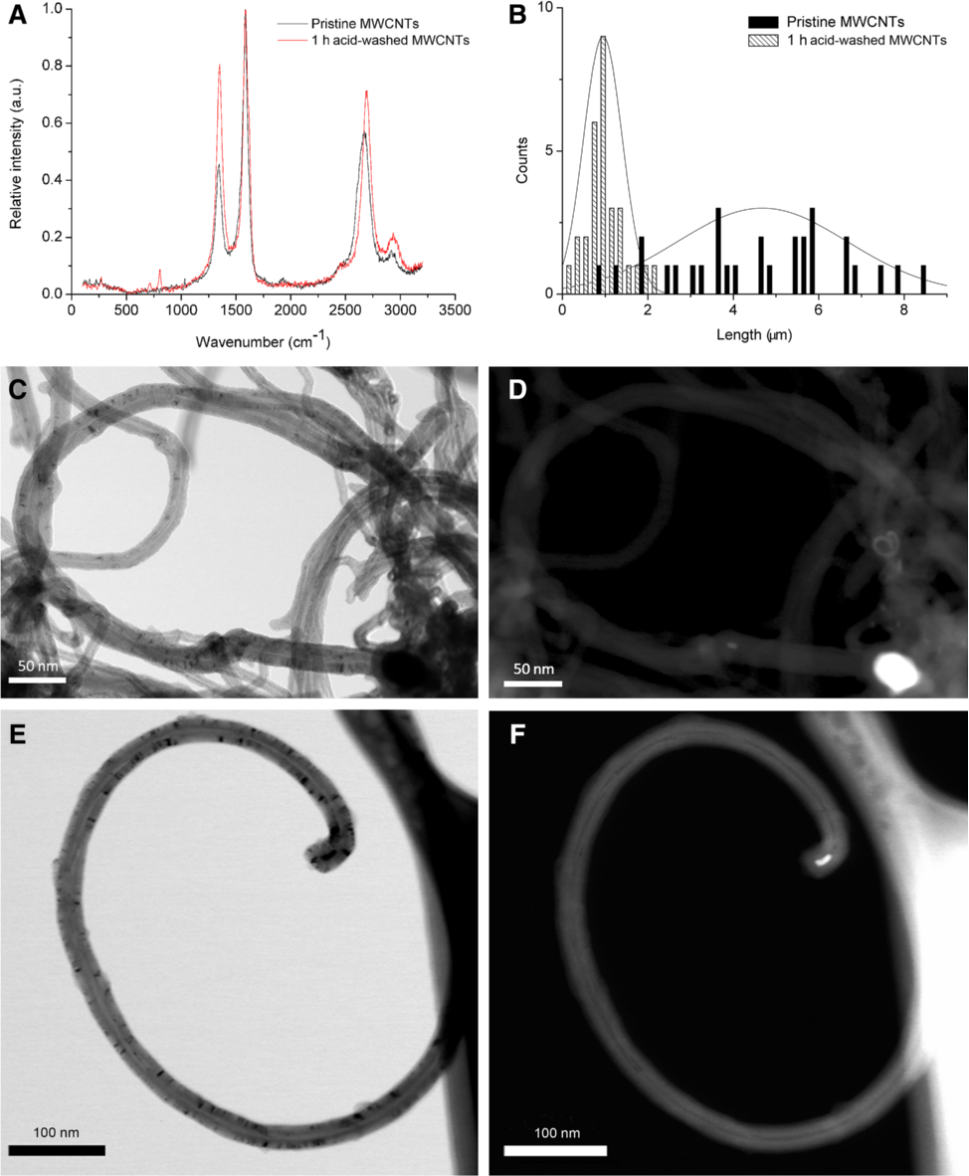
**Raman characterization, electron microscopy analysis and length distribution of MWCNTs.** Figure 1**A**. The figure is a histogram of the Raman spectra of pristine (black) and one hour acid-washed carbon nanotubes (red). Four independent bands have been identified for both samples, i.e., **D** band around 1350 cm-1, G band at 1585 cm-1, G’ band around 2690 cm-1, and an additional band around 2930 cm-1. Shifts in these bands are noticed for samples that have been treated with acid for 1 h. Figure 1**B**. Histograms of length distribution of pristine (a) and 1 h acid-washed MWCNTs (b) as identified by tapping mode Atomic force microscopy (AFM). At least 30 nanotubes have been analyzed for each one of the samples. Figure 1**C**, **D**, **E**, **F**: Figure 1**C** shows a representative bright-field image and Figure 1**D** shows the corresponding dark-field image of the MWCNT sample. The images demonstrated that the MWCNTs have a diameter of 10–20 nm and a typical multi-walled tubular morphology. Figure 1**D** shows representative dark-field STEM (DF-STEM) image of the native MWCNT sample that was acquired. The analysis demonstrated low amounts of the iron catalyst. Figure 1**E** shows a representative bright-field image and Figure 1**F** shows the corresponding dark-field image of the MWCNT sample. The dark-field image provides atomic number contrast information. The bright 10 nm particle at the end of the MWCNT in Figure 1**F** is a catalyst particle. Energy dispersive X-ray spectroscopy (EDS) showed that the catalyst particle was iron-rich. Further analysis of the MWCNT sample identified low amounts of the iron catalyst.

Raman spectroscopy was used to characterize the structure of pristine and acid-washed MWCNTs and to determine the degree of MWCNTs functionalization after acid treatment. Figure 1A shows the Raman spectra of pristine and acid-washed MWCNT. There are 4 bands identified in both pristine and acid-washed MWCNTs samples, i.e. D band around 1350 cm^-1^ that reflects the level of disorder in the sample, the G band around 1585 cm^-1^ indicative of the high degree order and well-structured samples, the G’ band around 2690 cm^-1^ representing the binary disordered band and lastly the peak around 2930 cm^-1^ indicative of the oxidation level of the sample being characterized. As shown, the D band was wider and had a higher frequency for the acid-washed sample when compared to the pristine MWCNTs. The shift in the D band indicates that the acid treatment minimally altered the chemical structure of MWCNTs by disrupting the structured walls and introducing additional functional groups (carboxylic acid groups) [53]. For the acid-washed MWCNTs there was also a shift of G’ band towards higher frequency; this may be due to the removal of metal catalysts, increase in the number of functional groups having electron accepting ability and decrease in the amorphous carbon. The ratio of intensity of D to G peaks indicate the degree of functionalization [54-56] and was 0.59 for pristine and 0.81 for 1 hr. acid-washed MWCNTs. This also confirms that the acid treatment increased the number of functional groups (i.e. free carboxylic acid groups) on the walls of the MWCNTs samples. Energy dispersive X-ray spectroscopy (EDX) confirmed the increase in the oxygen content due to the acid treatment and thus the increase in the MWCNTs degree of functionalization with free carboxylic acid groups as shown in Additional file 1. Further, the acid washing also reduced the catalyst content in the sample (Fe, 0.81). The content of the iron, cobalt and nickel were further analyzed by inductively coupled plasma-mass spectrometry (ICP-MS). Specifically, the MWCNT by ICP-MS contained 0.03% Fe ±0 .001, 0% cobalt, and 0% Nickel [57].

The length distribution of pristine and 1 h acid-washed MWCNT respectively is shown in Figure 1B (at least 30 individual MWCNTs were measured for each sample). AFM analysis showed that pristine MWCNT samples had an average length of 5499 ± 3009 nm while 1 h acid-washed MWCNTs had an average length of 825 ± 585 nm respectively indicating that acid treatment led to shortening of the nanotubes. The pristine and acid washed MWCNT had a diameter of 15 ± 5 nm. Moreover, acid washing also increased nanotube solubility in DMEM + FBS by two-fold compared to pristine MWCNT [58] as a result of the addition of the free carboxylic acid groups [2].

### Mitotic spindle disruption

**Figure 2:**
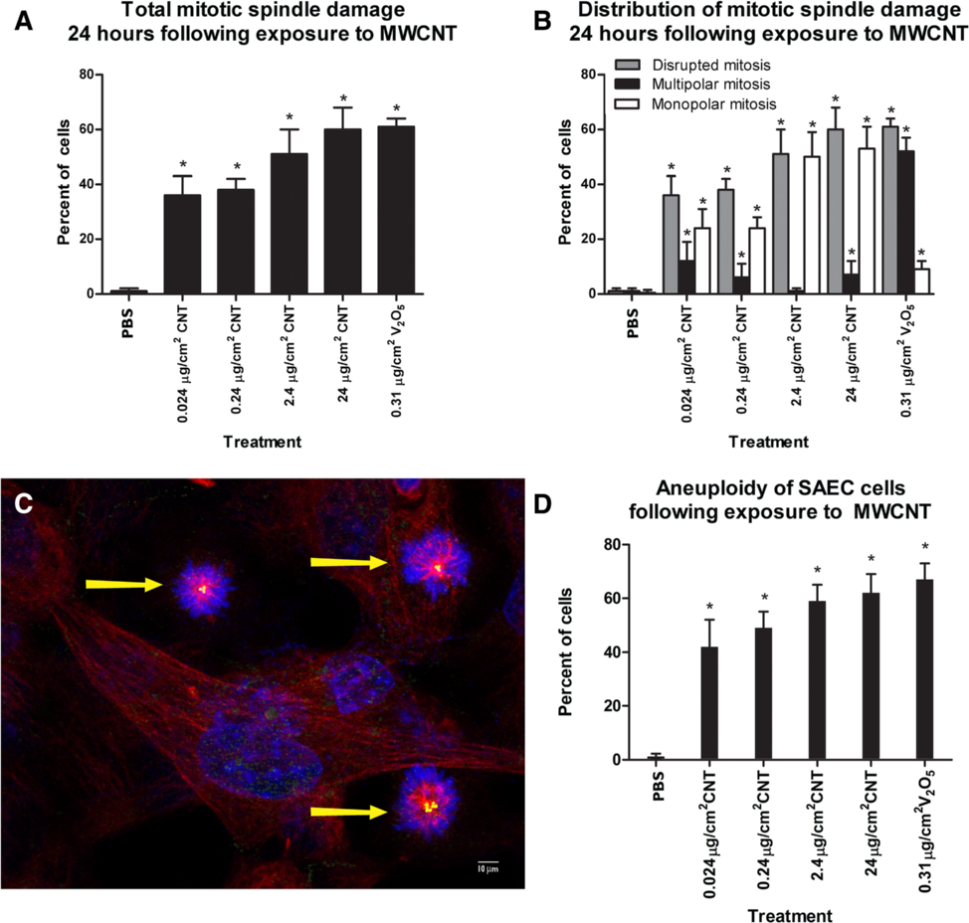
**Mitotic disruption following treatment with MWCNTs. A**: the bar graph demonstrates the mitotic disruption 24 hours following exposure to MWCNT. Mitotic spindle abnormalities are expressed as a percent of total mitotic figures. The abnormalities are separated into monopolar and multipolar mitotic spindles. The multipolar spindles include tripolar and quadrapolar mitotic spindles. *indicates significantly different from the unexposed control cells at p < .01; ± standard deviation. Figure 2**B**: The bar graph demonstrates the distribution of the mitotic spindle abnormalities in BEAS-2B cells following exposure to MWCNT. The white bars indicate the percent of mitotic cells with one mitotic spindle pole. The solid bars indicate the percent of total mitotic cells that had a multipolar mitotic spindle apparatus. The grey bars indicate the percent of mitotic cells with either a multipolar mitotic spindle or a monopolar mitotic spindle to show the percent of cells with any disruption of the mitotic spindle apparatus. *indicates significance at p <0.01.; ± standard deviation. Figure 2**C**: The photomicrograph of a culture exposed to 0.24 μg/cm^2^ MWCNT using a 40× objective. The yellow arrows indicate monopolar mitotic spindles. This figure demonstrates the typical monopolar phenotype of the cultures following exposure to MWCNT. Figure 2**D**: The bar graph demonstrates the percent of SAEC with an aneuploid chromosome number after a 24 hour exposure to MWCNT or the positive control V_2_0_5_. The solid bars indicate the level of apoptosis in the exposed and control BEAS-2B. The hatched bars indicate the level of apoptosis in the exposed SAEC. MWCNT exposure induced a dramatic elevation of chromosome loss and gain at all doses of exposure at levels equal to the positive control V_2_0_5_. *indicates significantly different from the unexposed control cells at p < .05.

Two human epithelial cell populations were examined to determine whether MWCNT induced genetic damage. Immortalized respiratory epithelial cells (BEAS-2B) were used to determine the effects of MWCNT on the mitotic spindle. Primary respiratory epithelial cells (SAEC) were included in the analysis to determine whether MWCNT induced errors in chromosome number. Treatment with acid-washed MWCNT induced a dose dependent mitotic spindle disruption (Figure 2A). The disrupted mitotic spindles were predominantly monopolar (Figure 2B). Figure 2C shows a 20× photomicrograph of the cultured cells with three monopolar mitotic spindles in one 40× field. Only 5-10% of the disrupted mitotic spindles were multipolar (Figure 2D).

### Chromosome number

Primary SAEC cells from a normal donor were used to investigate the effects of MWCNT on the chromosome number. The normal karyotype of the primary cells made it possible to evaluate the treatment related changes in chromosome number. FISH analysis for either chromosome 1 or 4 demonstrated a 2.25 ± 1.0% aneuploidy in the untreated SAEC cells (Table 1). The frequency of the cells with abnormal chromosome number is within the range reported in adult human cells in culture [59,60]. By contrast, the MWCNT-treated SAEC cells had a level of aneuploidy that was comparable to the vanadium pentoxide-treated positive control cells (Figure 2D; Table 1). Abnormal chromosome number was significantly elevated following MWCNT treatment as follows: 62 ± 7.0%, 24 μg/cm^2^; 59.0 ± 6.0%, 2.4 μg/cm^2^; 49 ± 6.0%, 0.24 μg/cm^2^ and 42 ± 10%, 0.024 μg/cm^2^ compared with control incidence of 2.25 ± 1.0%. Treatment with 0.31 μg/cm^2^ V_2_0_5_ resulted in 67 ± 6.0% aneuploid cells. The chromosome alterations in the MWCNT treated cells were predominantly gains of either chromosome 1 or 4 (Table 1). The chromosome losses accounted for 24%, 24 μg/cm^2^; 13%, 2.4 μg/cm^2^; 8%, 0.24 μg/cm^2^ and 12%, 0.024 μg/cm^2^. Chromosomal gains accounted for over 70% of the aneuploidy (Table 1). There was also a dose-dependent increase in the number of cells with gains of both chromosomes 1 and 4 indicating an increase in polyploid cells. The number of alterations of chromosome 1 was not statistically different than the alterations of chromosome 4, therefore; there was not a bias for a change of either chromosome.

**Table 1:** **Percent of chromosome errors in SAEC cells following treatment with MWCNT orV**_**2**_**0**_**5**_

**Dose MWCNT μg/cm**^**2**^	**Total % aneuploid cells**	**% loss of chromosome 1**	**% gain of chromosome 1**	**% loss of chromosome 4**	**% gain of chromosome 4**	**% gain of both chromosomes**
Diluent	2.25 ± 1.0	1.0 ± 1.0%	1.0 ± 1.0%	1.25 ± 1.0%	1.0 ±1.0%	0
0.024	42 ±10*	2.0 ± 1.26	15.0 ± 2.0*	3.0 ± 1.26*	16.4 ± 2.0*	12.0 ± 3.0*
0.24	49 ±6.0	1.7 ± 0.7*	23.7 ± 5.0*	2.0 ± 10*	25 ± 4.0*	18 ± 6.0*
2.4	59.0 ± 6.0*	3.4 ± 0.8*	26.0 ± 3.0*	4.3 ± 1.2*	25 ± 10*	23 ± 5.0*
24	62 ±7.0*	7 ± 3.0%*	49.3 ± 4.0%*	8.0 ±3.0%*	53.3 ± 5%*	44 ± 5.0%*
Dose vanadium μg/cm^2^						
0.31	69.0 ±7.0*	23.0 ± 5.0*	35.0 ± 9.0*	25.0 ± 11*	34.0 ± 7*	19.0 ± 6*

*Statistically significant at p < .05.

The distribution of the aneuploidy that was contributed by chromosome 1 and by chromosome 4 is detailed in the table as “Total % aneuploid cells”. The percent of cells with a gain in chromosome 1 and/or of chromosome 4 are indicated in the table under “Gain” of each chromosome. Cells with both chromosomes gained are indicated by “Gain of both chromosomes”. Cells with a loss of chromosome 1 and/or chromosome 4 are indicated in the table under “Loss” of each chromosome. *: p <0.05 of the treated cells compared to diluent control exposed cultures; ± standard deviation.

### Interaction of carbon nanotubes with mitotic spindle apparatus

**Figure 3:**
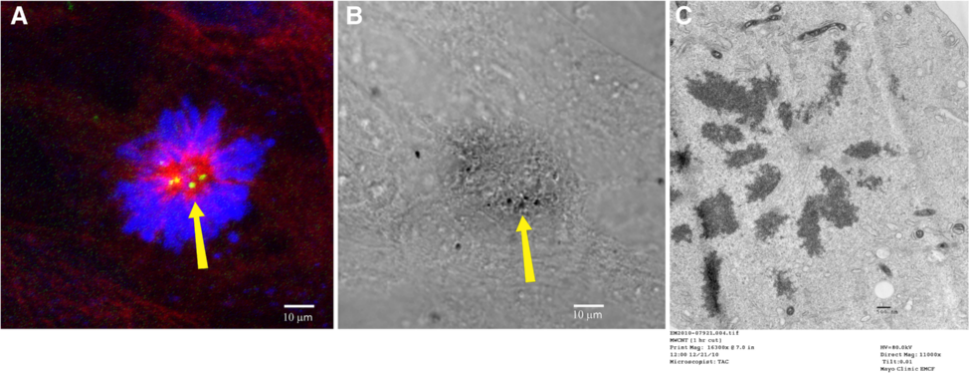
**MWCNT-treated cell with one spindle pole.** The photographs in Figure 3**A**-**C** show a monopolar mitotic spindle with one pole rather than the two poles which would be expected in a normal cell. The details of the detection protocol for the mitotic spindle components and the photography using the Zeiss Confocal are in the methods section. The tubulin in 3**A** was stained red using Spectrum red and indirect immunofluorescence. The DNA was detected by DAPI and was blue. The nanotubes were imaged using differential interference contrast and are black. In Figure 3**B**, the nanotubes can be seen in the nucleus, in association with microtubules, the DNA and the centrosome. Serial optical sections at 0.1 micron intervals using confocal microscopy confirmed the location of the nanotubes in the nuclear DNA and the tubulin including the microtubules of the mitotic spindle. Figure 3**C** is a high resolution TEM of a monopolar mitosis. The image was photographed at 11000× magnification.

**Figure 4:**
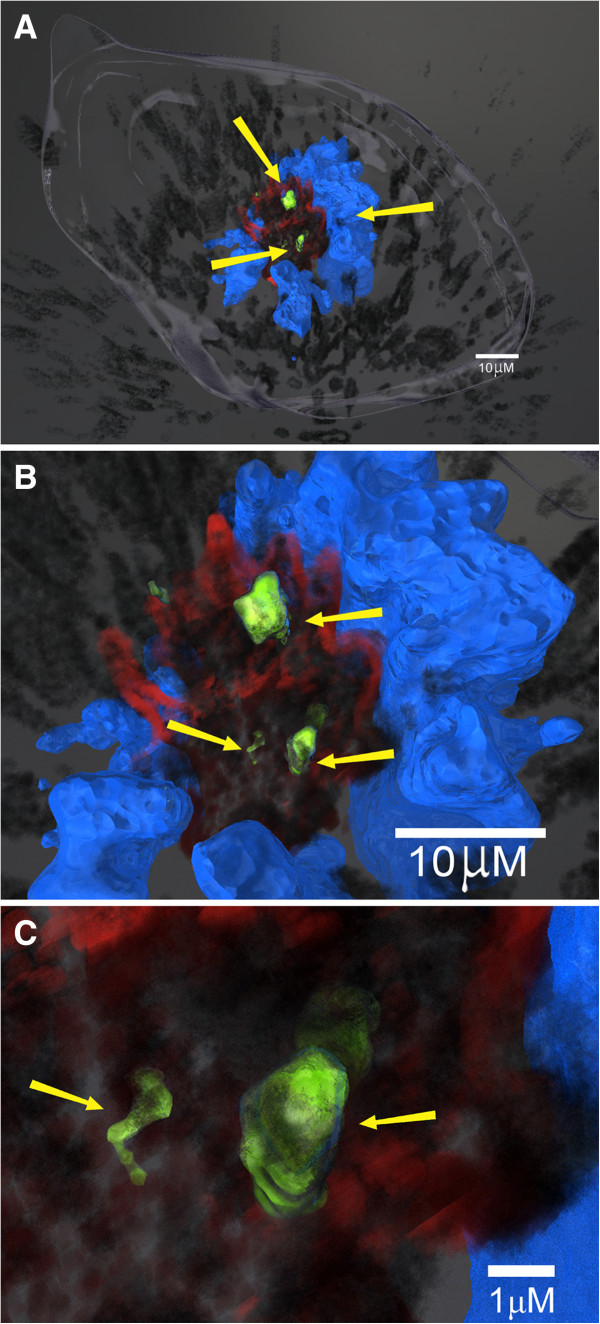
**A, B and C: Three-dimensional reconstruction of a MWCNT-treated mitotic cell. Figure**4**A**: This 3-dimension reconstruction was created from serial optical laser scanning confocal microscopy sections using immunofluorescence to identify centrosomes and microtubules while differential interference contrast was used to visualize aggregated MWCNT as previously described [24]. Briefly, nanotubes of 10 nanometers or greater could be visualized by their interference with transmitted light using DIC imaging. Because the nanotubes block the light, the nanotubes produce a black image. The reconstructed image shows aggregated nanotubes which appear as irregular tangled black structures located inside the cell in association with the centrosomes (green), the microtubules (red) and the DNA (blue). In this cell, the one spindle pole, the doughnut shaped DNA arrangement and the disruption of microtubule attachments to clustered centrosome fragments into a monopolar spindle apparatus suggest major perturbations in cell division. The yellow arrows indicate nanotubes in association with mitotic spindle and the DNA. Figure 4**B**: The yellow arrows indicate the nanotubes (black) in association with the centrosomes (green) and the microtubules (red). Figure 4**C**: The yellow arrows indicate nanotubes (black) inside the centrosome structure (green).

The MWCNTs were 10–20 nanometers in width. Nanotubes of 10 nanometers or greater can be observed using differential interference contrast imaging. MWCNTs were observed in the cytoplasm and the nucleus (Figure 3A). The MWCNTs also had a strong association with the centrosomes as shown in Figure 3B. The high frequency of monopolar mitotic spindles allowed confirmation of the monopolar phenotype by transmission electron microscopy (TEM) as shown in Figure 3C. The 3D reconstructed image demonstrates strong physical associations between the carbon nanotubes, the microtubules and DNA and the centrosomes (Figure 4A- B). The 3D reconstruction further demonstrated that MWCNTs not only associated with the centrosome but inside the centrosomal structure (Figure 4C).

### Viability and clonal growth

**Figure 5:**
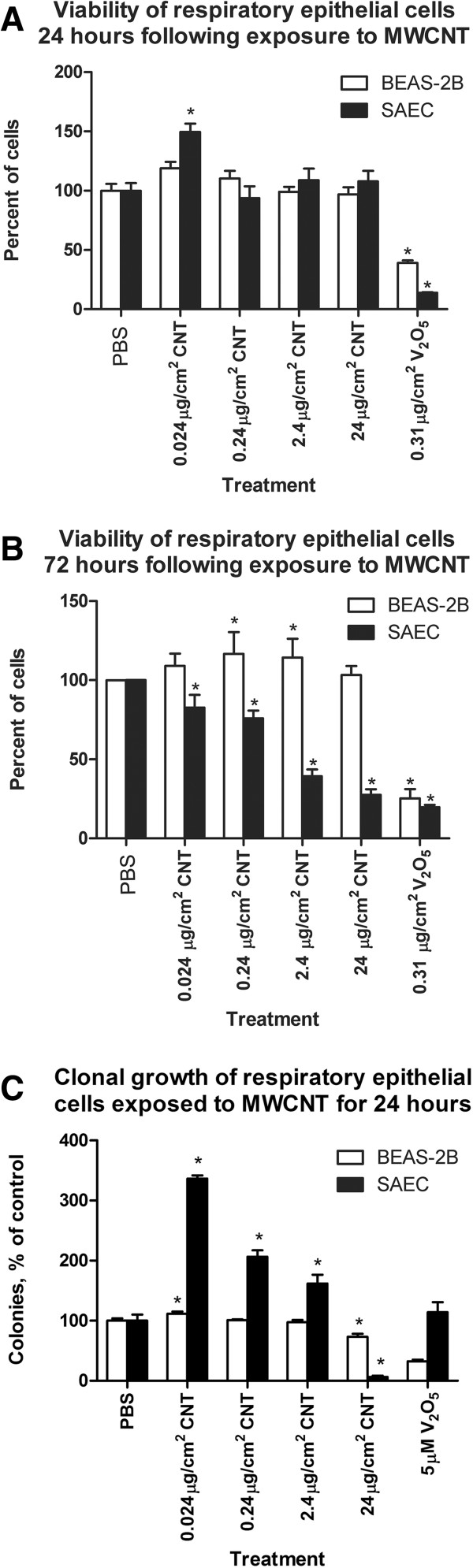
**A, B and C: Clonal growth and viability of BEAS-2B and SAEC cells.** Figure 5**A**: The bar graph represents viability of BEAS-2B and SAEC cells 24 hours following exposure to MWCNT or V_2_0_5._ The white bar indicates viability of BEAS-2B cells. The black bar indicates viability of SAEC cells. The viability was not reduced in either the BEAS-2B or the SAEC cells. Figure 5**B**: The bar graph represents the viability of BEAS-2B and SAEC cells 72 hours following exposure to MWCNT. The white bar indicates the viability of BEAS-2B cells and the black bar indicates viability of SAEC cells. MWCNT exposure resulted in reduced viability in the SAEC and the BEAS-2B at 0.024, 0.24, 2.4 and 24 μg/cm^2^ compared to control cells. The exposure to V_2_0_5_ resulted in reduced viability in SAEC treated cells at all doses. *indicates statistical significance of the treated cells compared to control cells at p <0.05. Figure 5**C**: The bar graph demonstrates the clonal growth in BEAS-2B cells 3 weeks following MWCNT exposure and SAEC cells 4 weeks following exposure. The black bars indicate the mean number of colonies of BEAS-2B cells and the white bars indicate mean number of colonies in SAEC cells. *indicates significance at p <0.05 of treated cells compared to control cultures.

Exposure to MWCNT did not reduce viability 24 hours after treatment in either the primary SAEC or the immortalized BEAS-2B cells (Figure 5A). Vanadium pentoxide treatment resulted in reduced viability in both SAEC and the BEAS-2B cells. Seventy-two hours following exposure, the viability of the SAEC cells was significantly reduced in cells exposed to 0.024, 0.24, 2.4 or 24 μg/cm^2^ MWCNT (Figure 5B). Three weeks following exposure, the BEAS-2B cells had a small increase in colony formation at 0.024 μg/cm^2^ (Figure 5C). One month following exposure, the SAEC cells had a reduced number of colonies at the highest dose; however, exposure to 0.024, 0.24 and 2.4 μg/cm^2^ resulted in a dramatic increase in colony formation (Figure 5C).

### Cell cycle

The impact of MWCNT-treatment on the cell cycle was evaluated by Click-iT EdU Flow Cytometry assay. Treatment with 24 ug/cm^2^ MWCNT induced a statistically significant increase in the percent of cells in S phase from 32.11% (PBS-treated) to 40.1% (Table 2). When the cells in G2 phase of the cell cycle were compared, exposure to the positive control, arsenic, resulted in 32.1% of the cells in G2 compared to 18.30% of the cells in the PBS control group thus indicating an arsenic-induced block in G2 (Table 2, p < .05).

**Table 2:** Distribution of the cell cycle in BEAS-2B cells 24 hours after treatment

**Treatment**	**% G1**	**% S**	**% G2**
24 hour PBS	43.25 ± 5.6	32.11 ± 6.5	18.30 ± 5.3
24 hour As	35.6 ± 6.9	26.38 ± 7.9	32.10 ± 6.7*
24 hour MWCNT	39.8 ± 4.0	40.1 ± 5.6*	15.90 ± 3.3

The table demonstrates the mean of percent of cells in G1, S and G2 phase of the cell division 24 hours following treatment with media, 5 μM arsenic or to 24 μg/cm^2^ MWCNT. The data is based on replicates of 6 that were repeated in 9 independent experiments.

*: p <0.05 of the treated cells compared to diluent control exposed cultures.

## Discussion

Since their discovery in 1991 [61] carbon nanotubes have been used for a variety of applications including fiber optics [62], conductive plastics, molecular electronics as well as biological and biomedical applications [63]. Although the durability and fiber-like structure of carbon nanotubes have raised concerns that carbon nanotubes may have effects similar to asbestos, the health effects have not been fully investigated [64,65]. Our data reported here are the first to show induction of monopolar mitotic spindles, aneuploidy, and a G1/S block in the cell cycle as well as a dramatic increase in colony formation following exposure to 10–20 nm diameter MWCNT. Exposure to 0.024 μg MWCNT/cm^2^ resulted in errors in chromosome number and mitotic spindle aberrations in greater than 40% of the cells examined. The dramatic increase in MWCNT-induced colony formation and aneuploidy observed in the primary SAEC cells was significantly higher than was previously observed in SWCNT-treated cells. The proliferation of cells with a high degree of genetic damage could result in the expansion of a population of genetically-altered cells. Cell proliferation is important in the second stage of pulmonary carcinogenesis, tumor promotion, while genetic instability is observed during the progression of preneoplastic cells to frank neoplasia [40,66]. During the progression of neoplastic disease, centrosome disruption is observed. The degree of centrosome disruption and aneuploidy is important because it is correlated with tumor stage [67-69].

The level of centrosome fragmentation, mitotic spindle damage and aneuploidy following MWCNT exposure was similar to the effects of the known carcinogen and positive control, vanadium pentoxide. MWCNTs were found in association with the DNA, the microtubules, the centrosomes as well as inside the centrosome structure. A previous investigation has shown that MWCNT are incorporated into the microtubules during polymerization thus forming a microtubule/nanotube hybrid [70]. The mitotic disruption that was observed following exposure to MWCNT may be due to a number of factors including incorporation of the nanotubes into the centrosome and microtubules of the mitotic spindle resulting in failed cytokinesis, failed centrosome duplication or inhibited centrosome separation. If two spindle poles are not formed during cell division, the chromosomes are not divided equally and chromosome errors occur.

Exposures that induce monopolar mitotic spindles produce daughter cells that fail to undergo cytokinesis and have double the number of chromosomes (polyploid) [71-73]. Although the data from the current investigation demonstrated that the aneuploidy was predominantly due to a gain of chromosomal material or polyploidy, the chromosomes were also lost in a significant number of cells suggesting that the genetic damage was due to more than a failure of cytokinesis. Asakura et al. [74] observed polyploid cells in cancer cell lines following exposure to 0.25 to 50 μg MWCNT of 80 nm diameter [74]. Although detailed analysis of chromosome loss and gain was not possible in a cancer cell line, the study demonstrated a significant number of polyploid cells which they attributed to a failure of cytokinesis. Carbon nanotubes have been observed in the bridge separating dividing cells [75]. Three dimensional reconstruction of MWCNT-exposed cells in the current study and of previously published SWCNT-exposed mitotic figures have shown carbon nanotubes integrated with the microtubules, the DNA and within the centrosome structure [19,24]. The disruption of cell division that has been observed following carbon nanotube exposure may be due to the incorporation of the carbon nanotubes into the microtubules that make up the division apparatus.

In this study, we observed fragmented centrosomes clustered into a single pole. These results are in sharp contrast to the multipolar mitotic spindles that have been observed with narrower SWCNT [19,20].

Centrosomes are duplicated in early G1/S of the cell cycle. The separation of the mother and daughter centrosomes by proteolytic enzymes is necessary for the exit from S phase and the formation of a bipolar mitotic spindle [76]. Incorporation of the stiff MWCNT into the centrosome may have resulted in a more rigid centrosomal structure which fractured during mitosis. In addition, the integration of the nanotubes into the centrosome structure could have prevented the proteolysis of the linker connecting duplicated mother and daughter centrioles in G1/S thereby preventing the centrosome separation necessary for the formation of a bipolar spindle [76]. Furthermore the excess of cells in the S phase and significantly lower number of cells in the G2 phase in the MWCNT-treated compared to the control cells in the current investigation indicate a G1/S block and a failure to progress to G2. Interaction of the nanotubes into the microtubules would potentially impact many cellular process including cellular transport of organelles (lysosomes, mitochondria, Golgi apparatus and endoplasmic reticulum), RNA and protein transport as well as phagocytosis and cell movement [77]. Kinesin and dynein motors move the organelles, chromosomes, proteins and RNA. Defects in the microtubule surface have been reported to result in detaching of the motors from the microtubule and interruption of cell signaling [77-80]. Aberrant cell signaling is a concern because it is important in the progression of carcinogenesis [81-83].

Although both SWCNT and MWCNT had a strong association with the microtubules that make up the mitotic spindle and induced aberrant mitotic spindles, the data suggests that the type of damage may be determined by the diameter of the carbon nanotubes. SWCNT of 1–2 nm in diameter [45], MWCNT of 5–10 nm [20] and the NanoLabs 10–20 nm MWCNT form hybrids with microtubules [70]. Both the SWCNT and the 10–20 nm MWCNT are incorporated into the centrosome structure. The stiffness of the nanotubes is determined by their diameter [84]. Although, carbon nanotubes have similar mechanical properties to the microtubules, the stiffness of the carbon nanotubes is a thousand-fold greater than that of the microtubules [84]. The incorporation of the more rigid MWCNT into the microtubules that make up the mitotic spindle fibers and the centrosome may reduce the elasticity of the mitotic spindle apparatus to a greater degree than the SWCNT. The elasticity of the mitotic apparatus is a critical factor in the separation of the centrosomes to organize two spindle poles as well as in the separation of the chromosomes during cell division [85].

Evidence from rodent exposure studies has demonstrated that high aspect ratio nanoparticles have carcinogenic properties [9,64,86,87]. Inhalation exposure is the route that most closely resembles occupational exposure. The lung is the principal target organ for carbon nanotube exposure [43]. The long thin carbon nanotubes induce inflammation, cell proliferation of type II epithelial cells and cellular atypia [30,31,33]. Recent investigations have shown that inhaled MWCNT migrate to the subpleural wall [44,88]. The fiber-like structure, evidence of carbon nanotube-induced inflammation, proliferation and cellular atypia in the lung as well as migration to the subpleural space, inflammation, macrophage injury and evidence of genotoxic damage have raised concerns that the material has carcinogenic properties similar to asbestos [44,64,89]. The lung as well as the parietal pleura is the sites of asbestos-induced carcinogenesis [64,90-93]. Injection of high doses of 100 nm diameter MWCNT into the abdominal cavity of p53 +/− mice has been shown to induce mesothelioma on the surface of the diaphragm [94]. In a more recent investigation of p53 +/− mouse exposure, Takagi et al. demonstrated a dose response of mesothelioma development after peritoneal injection of 3–300 micrograms of Mitsui-7 MWCNT [95]. Nagi et al. investigated the role of nanotube diameter in the development of mesothelioma in a rat model [96]. Greater inflammation and mesothelioma development were observed with the 50 nm diameter Mitsui-7 MWCNT of 10 microns or less in length compared to nanotubes of 145 nm diameter and similar length [96]. The mouse studies were criticized due to the route of exposure and the sensitivity of the genetically modified p53 knock-out mouse strain; however, the induction of mesothelioma was significant. The demonstration of mesothelioma at high exposures combined with our findings revealing disruption of the integrity of the division apparatus further suggest a carcinogenic potential for MWCNT. A manuscript in press by Sargent et al. has demonstrated that inhaled Mitsui-7 MWCNT material promoted the formation of lung adenocarcinomas in B6C3F1 hybrid mice following 3-methylcholanthrene (MCA) initiation [97]. While the data did not indicate tumor initiation by MWCNT, the exposure resulted in lung adenocarcinoma and adenoma in 90.5% of the mice exposed to MCA followed by inhaled MWCNT. The mouse lung tumors were large and 15% of the tumors were metastatic indicating tumor progression with some forms of MWCNT. Furthermore, the strong MWCNT-induced tumor promotion was observed in a hybrid mouse that is intermediate in sensitivity to lung cancer [98,99]. The exposure dose of the tumor promotion study of 32 μg/mouse is only 2.6 fold higher than the dose of the current *in vitro* investigation that shows significant chromosomal and mitotic spindle effects at the lowest administered dose of 0.024 μg/cm^2^[19]. Although lung cancer or mesothelioma have not been observed in humans exposed to MWCTs, centrosome disruption, aneuploidy and mitotic spindle aberrations as well as recent data indicating mesothelioma as well as lung tumor promotion and progression are a concern and indicate that caution should be used to prevent respiratory exposure to workers during the production or use of commercial products.

## Materials and methods

### Multi-walled carbon nanotubes acid washing

Multi-walled carbon nanotubes produced by chemical vapor deposition (Nanolab Inc. PD15L5-20) were acid-washed to remove iron catalyst. The MWCNT were suspended in a mixture of 3:1 v/v sulfuric acid (H_2_SO_4_) (96.4%, Fisher Scientific, Pittsburgh, PA): nitric acid (HNO_3_) (69.5%, Fisher Scientific, Pittsburgh, PA) for 1 hour in a water bath sonicator (Branson 2510, Fisher, Pittsburgh, PA) over ice. The mixture was subsequently diluted in deionized water (2 L) and filtered through a 0.2 μm polycarbonate membrane filter (Millipore, USA); the filtration step was repeated 6 times to remove catalysts or impurities. All cell exposure experiments were performed with one hour acid-washed MWCNT materials.

### Characterization of MWCNT

Atomic force microscopy (AFM) was used to investigate the length of both pristine and acid-washed MWCNT. Commercial Si tips (Asylum Research, AC240TS, USA) were used at their original resonance frequency, varying from 50 to 90 kHz. Pristine or acid-washed nanotubes (10 μg/ml) were deposited on mica surfaces (9.5 mm diameter, 0.15-0.21 thickness, Electron Microscopy Sciences, USA) and dried overnight under vacuum. Scans of 10 μm × 10 μm were acquired using tapping mode in air. At least 30 individual MWCNTs were analyzed to determine their length.

Raman spectroscopy was used to characterize the structure of both pristine and acid-washed MWCNTs. Raman analyses were performed at room temperature using a Renishaw InVia Raman Spectrometer (CL532-100, 100 mW, USA). The excitation source used an argon ion (Ar^+^) laser operating at 514.5 nm. MWCNT (pristine or acid-washed, 1 mg) were mounted on a clean glass slide (Fisher, Pittsburgh, PA) and a 20× microscope objective was used to focus the laser beam to a spot size of < 0.01 mm^2^ and to collect the scattered light. Low energy laser of < 0.5 mV and an exposure time of 10 sec were used to prevent unexpected heating effects of the MWCNT samples being analyzed. Detailed scans ranging from 100 to 3200 cm^-1^ were acquired.

The elemental analysis of the pristine and acid-washed carbon nanotubes was examined by energy dispersive X-ray spectroscopy (EDX). Both pristine and acid-washed MWCNT (1 mg/ml in water) were vacuum-dried on silica wafers. The experiments were performed using a Hitachi S-4700 Field Emission Scanning Electron Microscope (USA) and backscattered (BSE) electron detection in a single unit and operating at 20 kV.

ICP-MS) was performed to further analyze the chemical composition of the nanotubes as described previously. Carbon nanotubes were suspended in pure H_2_O (18.2 MΩ–cm) at a concentration of 1.0 mg/ml. One ml of each vortexed suspension was added to a 100 ml polytetrafluoroethylene digestion tube (CEM, Matthews, NC) along with 9.0 ml of ultrapure HNO_3_ and 1.0 ml of ultrapure H_2_O_2_ (Fisher Optima, Fisher Scientific, Pittsburgh, PA). Three replicate samples for each nanotube type were digested in the Microwave-Assisted Reaction System (CEM, Matthews, NC) by ramping up to 200°C for 15 min., holding at 200°C for 30 minutes, then cooling to 22°C, adapting a procedure as previously described [100]. There was no visible carbonaceous material remaining in any of the samples after digestion. After suspension (1 mg/ml), the metal content of the nanotubes was analyzed by ICP-MS using the Perkin-Elmer Nexion 300D [101], using ^54^Fe, ^60^Ni, and ^59^Co isotopes. Standards were certified multi-element standards in 1% HNO_3_.

### Dispersity analysis

The dispersity of pristine MWCNTs and acid-washed MWCNTs in Phosphate buffered Saline (PBS, Fisher, Pittsburgh, PA) was determined by centrifuging the corresponding suspensions (initial concentration 5 mg/mL for both pristine and acid-washed MWCNTs) at 3000 rpm for 5 min. Subsequently, 0.8 mL of the supernatant mixture was filtered through a 0.2 μm filter membrane. After complete drying under vacuum, the amount of pristine MWCNTs or acid-washed MWCNTs on the filter membrane was measured and the dispersity was calculated based on the starting volumes. The obtained values do not reflect the saturation dispersity.

### Cell culture

Two human respiratory epithelial cell populations were used to examine the potential genetic damage to MWCNT exposure. Immortalized human bronchial epithelial cells (BEAS-2B, ATCC, Manassas, VA) cultures of passage 4–6 were used to examine the mitotic spindle integrity. The high mitotic rate of the BEAS-2B cells allows examination of sufficient number of mitotic spindles following treatment. BEAS-2B cells grown in serum enriched media double every 18–20 hours and have normal mitotic spindle morphology. The high mitotic index of the BEAS-2B cells made it possible to analyze a sufficient number of mitotic spindles during the 24 hour exposure. Primary small airway respiratory epithelial cells (SAEC; Lonza, Walkersville, MD) from a normal human donor were used to determine the response of a normal cell population. In addition, the normal karyotype of the primary cells was essential for the examination of aneuploidy. The SAEC cells double every 20–24 hours which allowed analysis of a potential change in chromosome number and centrosome morphology of cells that have divided during the 24 hour exposure. The low mitotic index of the SAEC cells (0.5%) prevented the analysis of mitotic spindle integrity in this cell population. The BEAS-2B and SAEC cells were therefore analyzed 24 hours after exposure to allow a sufficient number of cells that have gone through division.

BEAS-2B cells were cultured in Dulbecco’s Modified Eagle Medium (DMEM) media supplemented with 10% serum (Invitrogen, Grand Island, NY). The SAEC cultures were cultured following manufacturer’s directions and using Cabrex media (Lonza, Walkersville, MD). The cell cultures were examined by electron microscopy and cytokeratin 8 and 18 staining to verify the epithelial phenotype of the cells as described previously [102].

### Treatment protocol

The immortalized BEAS-2B and the primary SAEC were exposed in parallel culture dishes to MWCNT or to the positive control, vanadium pentoxide (Sigma St. Louis, MO). Three independent experiments were performed for each exposure for SAEC and BEAS-2B respectively. MWCNT and vanadium control were suspended in media and sonicated over ice for 5 minutes and 30 minutes respectively. The cells were seeded in dishes and exposed 0, 0.024, 0.24, 2.4 and 24 μg/cm^2^ MWCNT or to 0.031 μg/cm^2^ vanadium pentoxide when the cells were 70% confluent. The one milliliter culture was treated with 0.024, .24, 2.4 and 24 μg/ml respectively. Twenty-four hours after exposure all cells were prepared for analysis of apoptosis and necrosis, integrity of the mitotic spindle, as well as the centrosome and chromosome number as described below.

### Viability and apoptosis

Triplicate cultures were prepared in 96 well plates (Becton Dickinson Franklin Lakes, NJ) for the analysis of viability using the Alamar Blue bioassay (Invitrogen, Carlsbad, CA), following manufactures directions as described previously [24]. Eight wells were performed for each treatment and dose. Three independent experiments were performed for the analysis of cellular toxicity by Alamar Blue. Parallel cultures were also prepared in duplicate in one milliliter chamber slides (Nunc Rochester, NY) for the analysis of apoptosis using the TUNEL assay following the manufacturer’s directions (Roche, Inc., Indianapolis, IN) with some modifications outlined previously [24]. A minimum of 100 cells were analyzed for each sample; experiments were repeated three times for a total of 300 cells for each treatment and dose, respectively for the analysis of apoptosis by the TUNEL assay. An additional positive control, 1.68 Molar DNase (Sigma St. Louis, MO) was used for the analysis of apoptosis. Twenty-four hours after dosing, cells in the chamber slides were fixed in 4% paraformaldehyde in phosphate buffer (Sigma St. Louis, MO) and stained with DAPI (Millipore Billerica, MA). The resulting stained samples were fluorescently analyzed using a Zeiss Axiophot fluorescent microscope (Carl Zeiss Microimaging Inc. Thornwood, NY).

### Mitotic spindle analysis

BEAS-2B was cultured in 1 milliliter chamber slides as described previously. Dual chambers were prepared for each treatment and each cell type. Three independent experiments were prepared for each cell type and treatment [24]. A minimum of 100 cells of good centrosome and mitotic spindle morphology were analyzed for each sample; experiments were repeated three times for a total of 300 cells for each treatment and dose, respectively. The centrosome integrity as well as the dispersion of carbon nanotubes in the cell cultures was evaluated The spindle integrity of the BEAS-2B cells was examined using dual-label immunofluorescence for tubulin and centrin to detect the mitotic spindle and the centrosomes, respectively. Primary rabbit anti-beta tubulin (Abcam, La Jolla, CA, USA) and mouse anti-centrin antibodies (a generous gift from Dr. Jeff Salisbury), and secondary Rhodamine Red goat anti-rabbit IgG and Alexa 488 goat anti-mouse IgG antibodies (Invitrogen, Carlsbad, CA) were used. The mitotic spindle and centrosome morphology were analyzed in the BEAS-2B cells using a laser scanning confocal microscope (LSM 510, Carl Zeiss MicroImaging Inc., Thornwood, NY) as previously described [103]. Briefly, a monopolar or multipolar mitotic spindle was counted as disrupted. The location of MWCNT was determined by differential interference contrast. Because the nanotubes block the light, the nanotubes produce a black image. To determine the association of the MWCNT with the microtubules of the mitotic spindle and the centrosome, serial optical slices was obtained to create a z-stack and permit three-dimensional reconstruction using LightWave software [104] by TEM following methods outlined previously [103]. Briefly, cells were fixed in 2% glutaraldehyde in sodium phosphate buffer, pH 7.2, for 2 h, postfixed in osmium tetroxide, dehydrated through an ethanol series, and embedded in Spurr’s resin (Sigma, St Louis, MO). Silver-gold sections were stained in 2% aqueous uranyl acetate and Reynolds’ lead citrate, observed using a JEOL 1200 EX electron microscope and recorded digitally.

### Chromosome number by fluorescence *in situ* hybridization (FISH)

Due to the necessity of a normal diploid karyotype for the analysis of chromosome number, the SAEC cells were prepared for analysis of the chromosome number. Fluorescence *in situ* hybridization (FISH) for human chromosomes 1 and 4 was used to determine the chromosome number (Abbott Molecular, Des Plaines, IL) according to the guidelines of the American College of Medical Genetics [105]. Three independent experiments for a total of 300 cells were evaluated for each treatment and dose. A minimum of 100 interphase cells of good FISH morphology were analyzed to determine the number of chromosome 1 and 4. The SAEC cells were photographed using a Zeiss Axiophot microscope and Genetix Cytovision software. Cells with three copies or greater than 4 copies of chromosome 1 or 4 were recorded as a gain for that chromosome. Cells with less than two copies of chromosome 1 or 4 were recorded as a loss of that chromosome. The loss and gain of both chromosomes were added to obtain the errors in chromosome number (aneuploidy).

### Colony formation

Triplicate cultures of SAEC cells were grown in T25 flasks. When the cells were 70% confluent they were treated with MWCNT. After 24 hours, the cells were trypsinized, counted and plated at 500 cells/well in 6-well plates for analysis of colony formation. One month following exposure, the cells were washed with PBS, stained with 10% crystal violet solution in neutral buffered formalin (Sigma, Saint Louis, MO) and colonies counted.

### Cell cycle analysis for DNA content

BEAS-2B cells were grown in six parallel T25 flasks. A total of 9 independent experiments were performed for the analysis of cell cycle. Twenty-four hours after exposure to 24 μg/cm^2^ MWCNT or to the positive control, 5 μM arsenic (Sigma, St Louis MO), the cells were washed twice with PBS and removed from the dishes with 0.25% trypsin prior to detection of the cell cycle. The cells were stained according to (Invitrogen) manufacturer’s instructions. EdU (5-ethynyl-2′-deosyuridine) is a nucleoside analog of thymidine and is incorporated into DNA during active DNA synthesis. Detection is based on a click reaction- a copper catalyzed covalent reaction between an azide and an alkyne. Twenty-four hours after exposure to MWCNT, the cells were washed twice with PBS and incubated with EdU for 2 hours to detect cells in S-phase. Following incubation, the cells were removed from the plate using 0.25% trypsin. After fixation and Click-iT Saponin permeabilization, CuSO_4_ was added to the cells to detect the EdU signal. The total amount of DNA was analyzed following incubation with 7AAD (7-aminoactinomycin D) using a LSR II flow cytometer (BD Biosciences Immunocytometry Systems, San Jose, CA). Data were analyzed and plotted using FlowJo v7.2.5 software.

### Statistical analysis

All analyses were performed using SAS/STAT (Version 9.3) for Windows. Chi-square analysis was used to determine statistical significance for the scoring of the mitotic spindle abnormalities and the number of cells with abnormal chromosome number. The number of viable and apoptotic cells were analyzed using analysis of variance (ANOVA). The mean of duplicate samples were used for the analysis. For cell cycle analysis, a mixed model ANOVA was used to compare the proportion of cells in G1, S and G2/M phase across treatment groups. Experimental block was utilized as a random factor. All differences were considered statistically significant at p < 0.05.

## Competing interests

The authors declare that they have no competing interests.

## Authors’ contributions

KJS contributed to the study design, writing of the manuscript, conducted experiments and analyzed FISH signals. LMS conceived of and designed the study, analyzed the experimental results and drafted the manuscript. SHR and MLK contributed to the experimental design, acquisition of funding and writing of the manuscript. MLK also analyzed the data for statistical significance. DTL contributed to the study design, conducted the experiments as well as contributed to the analysis of the data. CD acid washed the MWCNT and performed analysis of the material. AFH contributed to the study design and writing of the manuscript and acquisition of funding. JLS was involved in acquisition of funding and writing of the manuscript. DWP contributed to the study design and calculations of the dose for exposure. CZD contributed to the preparation and characterization of the MWCNT and drafting the description of the manuscript. MK performed ICP-MS and drafting of the manuscript. JM, KB, MS and JS contributed to writing of the manuscript and materials characterization. LC assisted with MWCNT characterization and photography, writing of the manuscript and preparation of the figures. All authors read and approved the final manuscript.

## Supplementary Material

Additional file 1 **Metal composition of Pristine and Acid-washed MWCNT.** Table: The table demonstrates the metal composition of the pristine and 1 hour acid-washed MWCNTs as measured by energy dispersive X-ray spectroscopy (EDX). Click here for file
