# Supplementary material for: Genotoxicity of multi-walled carbon nanotubes at occupationally relevant doses
Source: Part Fibre Toxicol. 2014 Jan 30;11:6. doi: 10.1186/1743-8977-11-6 (PMC3923549; doi:10.1186/1743-8977-11-6)
Supplement: Additional file 1 — Metal composition of Pristine and Acid-washed MWCNT. Table: The table demonstrates the metal composition of the pristine and 1 hour acid-washed MWCNTs as measured by energy dispersive X-ray spectroscopy (EDX). [file 1743-8977-11-6-S1.doc]

Additional file 1: Table S1. Metal composition of Pristine and Acid-washed MWCNT.

| **Element** | **Pristine**  **MWCNT (wt %)** | **1h Acid-Washed MWCNT (wt %)** |
| --- | --- | --- |
| C | 92.62 | 91.86 |
| O | 3.06 | 6.58 |
| Fe | 3.15 | 0.81 |
| Cu | 0.56 | 0.28 |
| S | 0.45 | 0.26 |
| Si | 0.16 | 0.22 |

Table: The table demonstrates the metal composition of the pristine and 1h acid-washed MWCNTs as measured by energy dispersive X-ray spectroscopy (EDX).
